# Ascites Cured by Pressure

**Published:** 1829-01-01

**Authors:** 


### XLII.

### CLINICAL INSTITUTION OF PARMA.

#### ASCITES CURED BY PRESSURE.

Our readers are aware that Sir Gilbert Blane proposed pressure on the head, for the cure of hydrocephalus, and that the plan has been adopted by some others —not, we should suppose, with great success, otherwise we should have heard of it. Anasarca of the legs is every where treated by pressure, in the form of bandages, to promote absorption—or, rather perhaps to force the serous infiltration into another and worse locality. Indeed, we have generally observed, that patients have complained of oppression about the chest, or sense of fulness in the abdomen, when tight bandages were applied to prevent or remove anasarcous swellings. Till the secretions and excretions are set free, there is little permanent relief obtained in dropsy of any kind. The principle of pressure has been applied in the following case of ascites, by Dr. Speranza, physician to the Clinical Institution of Parma.

*Case.* A female had laboured for some months under considerable effusion into the cavity of the abdomen, the consequence of peritoneal inflammation, which had succeeded to a laborious accouchment. She entered the Parma Clinical Institution in the month of April, in a very weak condition, being apparently worn down by a slow fever, and lactation beyond her powers. The digestive functions were very much deranged—the urine scanty and turbid—thirst—constipation. The great distention of the abdomen by effusion, prevented any accurate examination of the viscera, as to their state of soundness. There was general emaciation, and a highly cachectic aspect. After a fair trial of diuretics, purgatives, mercurials, and the usual remedies, Dr. Speranza declined to recommend paracentesis abdominis, from a conviction, that this operation is never of any ultimate service; he had recourse, therefore, to the bandage of Monro. Under the influence of the pressure thus produced, the urine became copious, so that, at length, it amounted to *fifteen pints daily!* In the course of three weeks, the abdomen was reduced to its natural size, and there was no further effusion. The sulphate of iron, squills, and a nourishing diet contributed to a completion of the cure. She was ultimately discharged from the Hospital in perfect health.

It would be a very curious fact, if proved by subsequent experience, that pressure on a distended abdomen caused an increase of the urinary secretion. We could only account for it in this way:—the pressure augmented the action of the absorbents, or checked the activity of the exhalents, or both—and the blood-vessels being replete with serous fluids, the secretion of the kidneys was augmented, and thus the ascites cured. We think the remedy, especially if combined with *diuretic frictions,* might be well worthy of trial.

